# Some Contributions for Antenna 3D Far Field Characterization at Terahertz

**DOI:** 10.3390/s21041438

**Published:** 2021-02-19

**Authors:** Laurent Le Coq, Nicolas Mézières, Paul Leroy, Benjamin Fuchs

**Affiliations:** Institut d’Electronique et des Technologies du Numérique (IETR), UMR CNRS 6164, University of Rennes I, F-35042 Rennes, France; Laurent.Le-Coq@univ-rennes1.fr (L.L.C.); nicolas.mezieres@univ-rennes1.fr (N.M.); paul.leroy@univ-rennes1.fr (P.L.)

**Keywords:** antenna measurements, terahertz (THz) antenna, circular polarization

## Abstract

The three-dimensional (3D) characterization of antenna far field patterns at terahertz frequencies is addressed. This task is challenging, because the phase of the electric field is difficult to measure accurately and reliably. From the sub-millimeter wave range, the small wavelength indeed significantly increases the impact of mechanical and electrical errors. Models and procedures to estimate these errors are proposed to mitigate their effects. The 3D far field patterns of a circularly polarized horn measured at 300 GHz and a multibeam pillbox antenna at 270 GHz are shown. The agreement between the 3D measurements and the two-dimensional (2D) patterns of reference as well as the radiated pattern before and after correction demonstrates the interest of the proposed approach and experimentally validate the proposed error estimation procedures. The methodology can be applied to direct far field measurement facilities as well as compact antenna test ranges.

## 1. Introduction

The characterization of antenna radiation patterns at terahertz frequencies is a challenging task. It imposes stringent constraints on traditional antenna measurement techniques in terms of mechanical tolerances, positioning accuracy, thermal stability, and so on in order to obtain reliable measurements.

Several terahertz antennas have been successfully characterized using a direct far field measurement setup, as reported in [1,2,3,4,5,6,7,8]. In most of these examples, the measured patterns are two-dimensional (2D). However, three-dimensional (3D) measurements are meaningful when considering antennas with specific patterns (with a beam tilt of a frequency beam scanning behaviour for instance).

Let us point out that, at terahertz frequencies, the far field distance quickly becomes very large [9]. As a representative example, the far-field distance for an Antenna Under Test (AUT) of maximum dimension 10 cm is already 20 m at 300 GHz. Compact Antenna Test Ranges (CATRs) are a good alternative to overcome the limitation of AUT dimensions. They enable to generate far-field conditions in indoor ranges, even at terahertz frequencies. The CATRs are composed of a feed (typically a horn) that is associated to a collimating element to produce a plane wave. The AUT is then placed in the so-called quiet-zone, where the peak-to-peak variations of the field in amplitude and phase are typically below ±0.5 dB and ±5°, respectively. The focusing system of CATRs can be a reflector [10], a lens [11,12] or a hologram [13,14,15]. The major challenges of CATRs for terahertz measurements are the accurate manufacturing of the large collimating element and the verification of the quiet-zone field phase specifications.

Besides, near field scanning, and mainly the planar version, has been successfully used to measure the radiation pattern of terahertz antennas [16,17,18,19,20,21]. The amplitude and phase of the AUT near field are sampled with a probe antenna and the far field characteristics are derived while using numerical methods. The sampling area has to be sufficiently large, so that most of the energy that is transmitted by the AUT is captured. Moreover, the field sampling must be dense enough to satisfy the Nyquist sampling criterion of half a wavelength. The main error source of planar near field scanning systems are the phase errors that often stem from an inaccurate planar movement of the probe antenna. The uncertainties coming from flexing and bending cables and the reflections between the probe and AUT may also impact the accuracy of the measured phase and, therefore, induce errors in the far field characterization.

This paper addresses the characterization of antenna far-field patterns at terahertz. The practicalities and main sources of measurement errors encountered when measuring antennas at such high frequencies are reviewed. A special focus is brought on the rotary joints and cable-related errors, for which a correction method is described and the effects are shown on far-field measurements. The methodology can be applied to both direct far field measurement facilities and CATRs.

The paper is organized, as follows. The measurement of antennas at terahertz and its inherent sources of errors are reviewed and a procedure for compensating the error introduced by the rotary joint in the emission part is detailed in Section 2. This method is applied and experimentally validated by the 3D characterization of two radiating structures in Section 3. Conclusions are drawn in Section 4.

## 2. Antenna Measurement Analysis and Assessment

### 2.1. Sources of Measurement Errors

**Figure 1:**
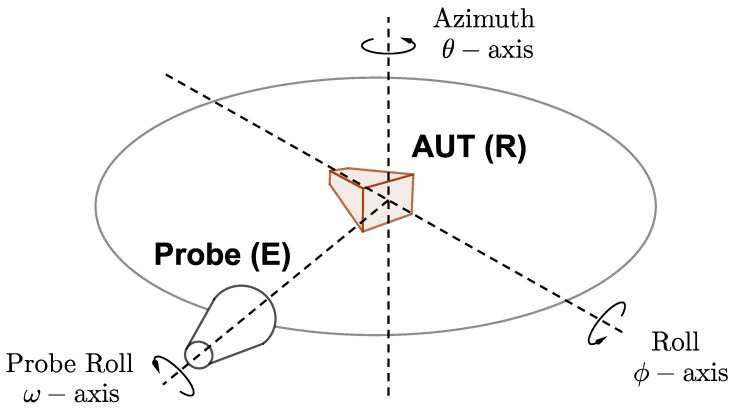
Measurement positioning system scheme with its rotation axis. The rotation angles θ and ϕ allow moving the probe along a sphere centered around the AUT and ω is used to a mechanically change the probe polarization direction.

Let us first describe our far-field antenna measurement setup, for which a representation is shown on Figure 1. We use a classical positioning system configuration: the AUT is placed on a roll (ϕ) over slide over azimuth (θ) positioner. The emitting probe is placed on a roll motion axis (ω) and it transmits a 27 dBi linearly polarized field.

In this configuration, the measured data are corrupted by three main kinds of errors:

Stray signals errors: the different impacts of multiple reflections between the AUT and the feed probe, between the AUT/probe and the setup/receiver, and the room scattering.

Electrical errors: the errors that are introduced by the RF system, such as its dynamic range, its non linearity, the effects of rotary joints and flexible cables, leakage, cross-talk, and thermal drift.

Mechanical errors: concerns both the emission and reception parts, and, more specifically, the error of positioning and orientation of each part with respect to the other one.

At terahertz, and more specifically from the sub-millimeter wave range, an accurate phase measurement is the key issue to properly characterizing 3D radiation patterns. The accuracy of the positioning systems and the stress applied on the connection chain between the frequency extenders and the VNA are the main error contributors. On the emission part, the whole frequency extender (VDI TxRef type) is rotating, which induces stress on three coaxial cables (RF signal, LO signal, and Reference IF signal). On the reception part, the receiver (VDI Rx type) is localized on the roll axis, and then rotation is as well applied to this module. However, as a triplexer is added, only one cable is used for DC, LO and IF signal. As an illustrative example, a distance variation of 0.1 mm between the probe and AUT induces at 300 GHz a phase variation of the transmitted signal of 36°. Therefore, our efforts are focused on both the mechanical errors and the uncertainties due to the rotary joints and flexible cables. Note that the effects of joints and cables can be merged as flexible cables enable the rotation in absence of rotary joint.

### 2.2. Qualitative Analysis of Errors

Generally speaking, mechanical and/or electrical errors induce phase errors. These errors must be analyzed according to the location of each rotation axis in the overall positioning system, see Figure 1.

#### 2.2.1. Emission Part

On one hand, there are electrical errors that are generated by the cable stress during rotation. On the other one, mechanical ones are produced by the combination of the pointing error, the offset of the probe, and the wobble of the axis. The importance of the latter depends on the distance between the rotation axis and the probe reference point. In general, the eccentricity is very small and it can be neglected. In our case, this eccentricity is smaller than ±2μm.

**Figure 2:**
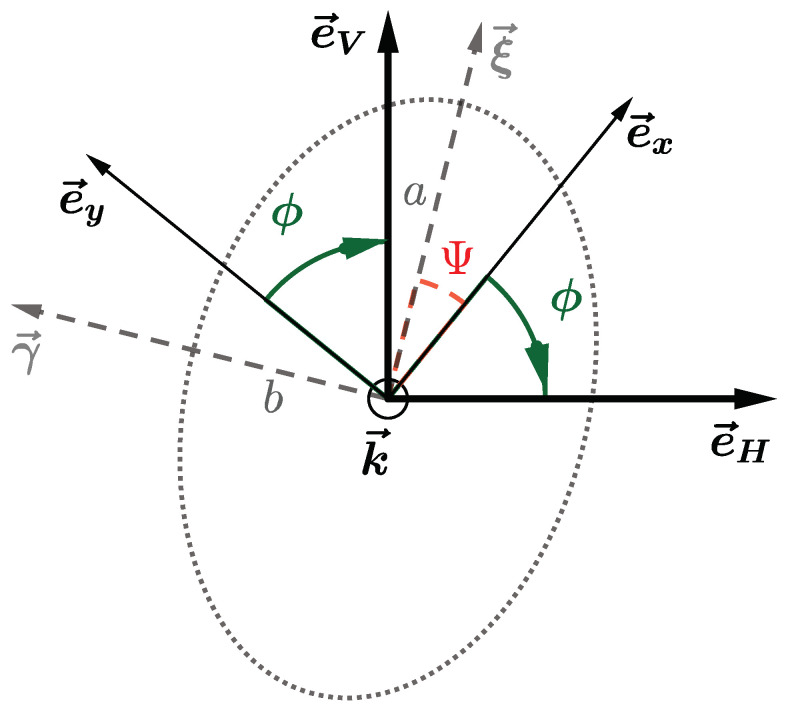
Polarization ellipse and coordinates systems at θ=0 degree: the major axis *a* and minor axis *b* are along vectors $\vec{\xi }$ and $\vec{\gamma }$ respectively.

The overall inaccuracy depends on the emission rotation angle ω, as represented in Figure 1. This uncertainty can be seen as a generalized channel imbalance error written RJE(ω), where RJE stands for Rotary Joint Emission. Such error impacts the field reconstruction, as it corrupts the reconstruction of the polarization ellipse. It means that an undesirable, but fixed phase shift is applied between the two components of the measured field. As a consequence, the definition of the tilt angle (angle Ψ in Figure 2) used for Ludwig 3 representation and the decomposition in Left and Right Hand Circular Polarizations, (LHCP) and (RHCP), respectively, are corrupted.

#### 2.2.2. Reception Part

Two rotation stages have to be considered: the roll axis (rotation of angle ϕ) and the azimuth axis (rotation of angle θ), as represented in Figure 1. The electrical error is generated by the cable stress and the transmission characteristics of the rotary joints, depending on the rotation angle for each axis. The mechanical error is produced by a combination of wobble, eccentricity, and distance between the rotation plane and the observation point. Note that the azimuth axis errors are only θ-dependent, whereas the roll axis errors are mainly ϕ-dependent, provided that the eccentricity can be neglected. As a result, the overall error can be considered independently for each axis. These two functions are denoted ${\mathrm{RJR}}_{az}\left(\theta \right)$ and ${\mathrm{RJR}}_{roll}\left(\phi \right)$ for the azimuth and roll axis, respectively, where RJR stands for Rotary Joint Reception.

The roll axis error induces a variation of the phase according to ϕ at the pole (θ=0°). This non-physical representation leads to an erroneous spherical harmonic spectrum.

The azimuth axis error induces a phase variation according to θ only. It might lead to the identification of spherical modes of higher degrees and/or orders than expected. This corruption of the measured signal can be seen as a complex ripple, whose periodicity, according to ϕ, depends on the sampling strategy.

#### 2.2.3. Summary

All of these errors have various effects on the spherical harmonic spectrum and, therefore, on the field reconstruction. At first, the non-uniqueness of the electrical field at the pole (θ=0°) should be mitigated, since it inevitably leads to a non-physical representation. Furthermore, the channel imbalance must be carefully corrected, since it affects all of the data points and the computations involving the use of two orthogonal components of the field (e.g. axial ratio). Finally, the errors, depending on the azimuth angle θ, strongly corrupt the spherical mode determination: they induce unexpected ripples on the 3D field reconstruction.

### 2.3. Error Model

The measured scattering parameter $S_{21}^{\mathrm{meas}}$ can be expressed as a function of the true, uncorrupted, one $S_{21}$, as follows:

(1) $$\begin{array}{cc} S_{21}^{\mathrm{meas}}\left(\theta ,\phi ,\omega \right)\; & \approx S_{21}\left(\theta ,\phi ,\omega \right){\mathrm{RJR}}_{az}\left(\theta \right){\mathrm{RJR}}_{roll}\left(\phi \right)\mathrm{RJE}\left(\omega \right)\\ \; & \approx {\tilde{S}}_{21}\left(\theta ,\phi ,\omega \right){\mathrm{RJR}}_{roll}\left(\phi \right)\mathrm{RJE}\left(\omega \right). \end{array}$$

The error functions, depending on ϕ and ω, should be first considered. Accordingly, the compensated signal ${\tilde{S}}_{21}\left(\theta ,\phi ,\omega \right)$ is corrupted by a residual error that is only θ-dependent, which means that the electric field polarization is properly estimated at each observation point. This remaining error is mainly due to both the rotary joint and the effect of the wobble of the azimuth stage. It mainly produces a continuous error on the phase, as the magnitude variation is low. As a consequence, it can be seen as a combination of a misalignment of the AUT in the general coordinate system and spurious effects.

### 2.4. Estimation of Rotary Joint Coefficients

#### 2.4.1. Case of Two Linearly Polarized Antennas

The estimation of the probe channel imbalance and the rotary joints at the reception (${\mathrm{RJR}}_{az}$ and ${\mathrm{RJR}}_{roll}$) is addressed in [22,23]. It can be achieved by measuring the transmission coefficient between two linearly polarized antennas (the probe and an antenna) while using a ϕ sampling range at the pole θ=0 for two orthogonal polarization orientation of the probe ω. The error can then be estimated, as the uncorrupted signal must be either a cosine or a sine function of ϕ. Although efficient, this procedure requires using a linearly polarized antenna in order to enforce the trigonometric expression of the signal. Consequently, the characterization of a circularly polarized antenna needs a first measurement in order to estimate the mechanical error. In the sub-millimeter wave range, this antenna substitution turns out to be a difficult task because of the unavoidable mechanical inaccuracy.

#### 2.4.2. Case of Mixed Polarized Antennas

The AUT can be used to determine the mechanical error, depending on ω and ϕ angles without resorting to an additional user handling, thus avoiding the main drawback of the previous procedure. Even if the measurement process is the same, the AUT polarisation is not known, and we can no longer assume that the measured signal is of cosine and sine types. Another strategy of processing is proposed.

We consider the local coordinate system $\left(\vec{e_{x}},\vec{e_{y}}\right)$ that is tied to the AUT for a probe located at θ=0, as illustrated in Figure 2. Using the notations from this figure, let $\vec{\xi }$ and $\vec{\gamma }$ be the vectors defining respectively the major axis *a* and minor axis *b* of the polarization ellipse. The axial ratio is thus a/b. These ellipse axis are tilted by some angle Ψ according to $\left(\vec{e_{x}},\vec{e_{y}}\right)$. Finally, the vectors ${\vec{e}}_{H},{\vec{e}}_{V}$ define the direction in which the field is measured, depending on the horizontal or vertical orientation of the probe, respectively. Figure 2 shows all of these notations.

The field can be expressed using the vectors defining the polarization ellipse:

(2) $$\vec{E}=E_{0}\left[\alpha_{\mathrm{LHCP}}\left(\vec{\xi }+j\vec{\gamma }\right)+\alpha_{\mathrm{RHCP}}\left(\vec{\xi }-j\vec{\gamma }\right)\right].$$

where $\alpha_{\mathrm{LHCP}}$ and $\alpha_{\mathrm{RHCP}}$ are the polarization coefficients. Because of imbalance between horizontal and vertical orientation of the probe, a complex factor RJE is introduced, as explained in Section 2.2.1. The representation of the complex field in the $\left(\vec{e_{H}},\vec{e_{V}}\right)$ basis verifies $E_{H}=\vec{E}\cdot {\vec{e}}_{H}$, for measurement with the horizontal orientation, and $E_{V}=\mathrm{RJE}\;\vec{E}\cdot {\vec{e}}_{V}$ for the vertical one. In addition, the measured components, $E_{V,\mathrm{meas}}$ and $E_{H,\mathrm{meas}}$, are also corrupted by the reception rotary joints ${\mathrm{RJR}}_{roll}$ and ${\mathrm{RJR}}_{az}$, meaning that:

(3) $$\begin{array}{cc} E_{H,\mathrm{meas}} & ={\mathrm{RJR}}_{az}\left(0\right){\mathrm{RJR}}_{roll}\left(\phi \right)\vec{E}\cdot {\vec{e}}_{H}\\ E_{V,\mathrm{meas}} & ={\mathrm{RJR}}_{az}\left(0\right){\mathrm{RJR}}_{roll}\left(\phi \right)\mathrm{RJE}\;\vec{E}\cdot {\vec{e}}_{V} \end{array}$$

Their influences can be mitigated by working on a specific ratio *R*, which reads using (2):

(4) $$R\left(\phi \right)=\frac{E_{H,\mathrm{meas}}-\frac{j}{\mathrm{RJE}}E_{V,\mathrm{meas}}}{E_{H,\mathrm{meas}}+\frac{j}{\mathrm{RJE}}E_{V,\mathrm{meas}}}e^{-2j\phi }=\frac{\alpha_{\mathrm{LHCP}}}{\alpha_{\mathrm{RHCP}}}e^{-j2\Psi }.$$

Because the probe is located at the pole θ=0, the ratio *R* is constant with respect to ϕ. By doing multiple measurements along this rotation axis at positions $\phi_{m}$, m=1,⋯,M, we can make independent measurements of this ratio, namely $R:={\left(R\left(\phi_{m}\right)\right)}_{m=1}^{M}$. We propose estimating the coefficient RJE by minimizing the variance of the sample R. An unbiased estimator of the variance is given by:

(5) $$\mathrm{Var}\left(R\right)=\frac{1}{M-1}\sum_{m=1}^{M}{\left|R\left(\phi_{m}\right)-\bar{R}\right|}^{2}$$

where $\bar{R}$ is the sample mean. The minimization of this variance is achieved by a standard gradient descent algorithm that considers the complex variable RJE as a 2D parameter.

**Figure 3:**
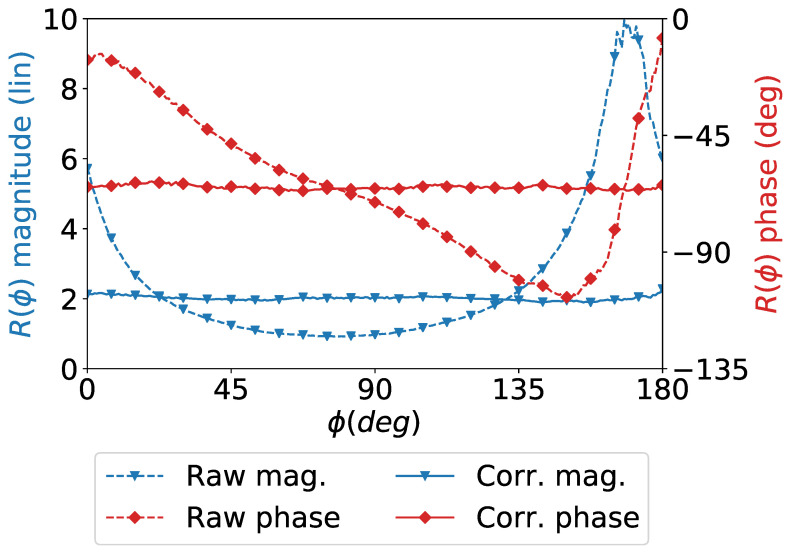
Measured and corrected ratios *R* in (4) for the horn antenna at 300 GHz presented in Section 3.2. Raw curves (dotted) show the ratios with no correction (RJE = 1) and the corrected ones (plain) with the RJE computed using the described approach.

This procedure is applied on measurements that were carried out at 300 GHz. The RJE is estimated and the ratio *R* is corrected accordingly. Figure 3 shows the results before and after correction over the ratio *R*. The cutting planes, shown later in Figure 8 further demonstrate the impact of this correction.

The programming languages Python (https://www.python.org/) and Julia (https://julialang.org/) have been used to implement the procedures.

## 3. Antenna Far Field Measurements at Terahertz

The previously described methods are applied in order to reduce the potential sources of errors as much as possible. The characterization of two radiating structures is shown after describing our 3D radiation pattern measurement procedure.

### 3.1. Antenna Measurement Procedure

#### 3.1.1. 3D Pattern Characterization

The 3D radiation pattern measurement duration for antennas at terahertz can be prohibitive because of the required sampling of the sphere. As detailed in [22,24], the use of spherical harmonics imposes to sample the sphere with an equi-angular step: Δθ=Δϕ≈λ/(2a), with *a* being the radius of the smallest sphere enclosing the AUT. The 3D pattern measurement at 300 GHz of an AUT of maximum dimension 2a=10 cm for the measurement of the 3D pattern requires a sampling step of less than 0.6, which leads to a number of sampling points greater than $2\times 10^{5}$ for each polarization of the field.

Strategies have been recently proposed in the microwave frequency range to significantly lower this number of sampling points and, thereby, reduce the characterization duration [25,26,27]. The main steps of the procedure are the following:

- Deriving the spherical harmonic truncation order $N_{t}$ from the largest electrical dimension of the AUT: $N_{t}=\left[ka\right]+10$.
- Sampling the sphere according to an “igloo” discretization: Δϕ=Δθ/sinθ, such that the total number of sampling points is approximately equal to $2/3\times {\left(N_{t}+1\right)}^{2}$, which means less than $1.1\times 10^{5}$ points for the considered example.
- Reconstructing the spherical mode coefficients using any readily available sparse recovery algorithm.
- Interpolating the field over the sphere.

More details about this procedure are available in [26].

#### 3.1.2. Validation Methodology

The goal is to assess the measurement itself. For that purpose, we perform two independent measurements in the same configuration and with the same systematic errors corrupting the dataset. First, a 3D measurement is carried out following an igloo sampling strategy that is coupled with a sparse spherical harmonic expansion. Subsequently, standard Nyquist measurements of 2D radiation patterns are performed with a small angular step in order to provide a reference for validating the 3D far field reconstruction.

### 3.2. Circularly Polarized Horn at 300 GHz

**Figure 4:**
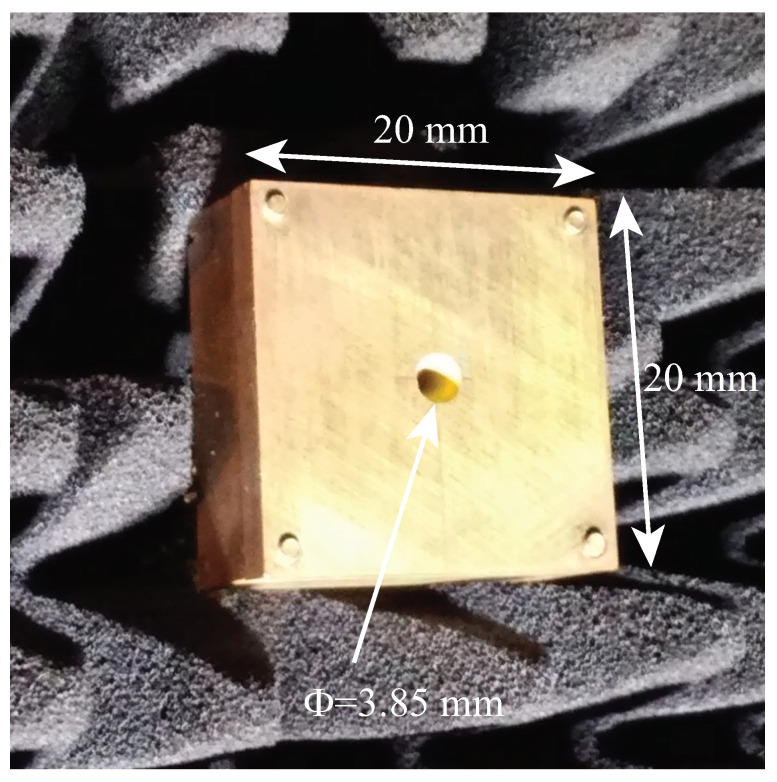
Picture of the circularly polarized horn antennas designed to work in the 270–330 GHz frequency range.

The AUT is a circular horn coupled with a polarising feeder, a cross of length 500 μm and width 90 μm. This antenna, as shown in Figure 4, is an in-house prototype designed to achieve a 18 dBi directivity. The circularly polarized horn is manufactured in a 20 mm × 20 mm × 20 mm brass volume.

**Figure 5:**
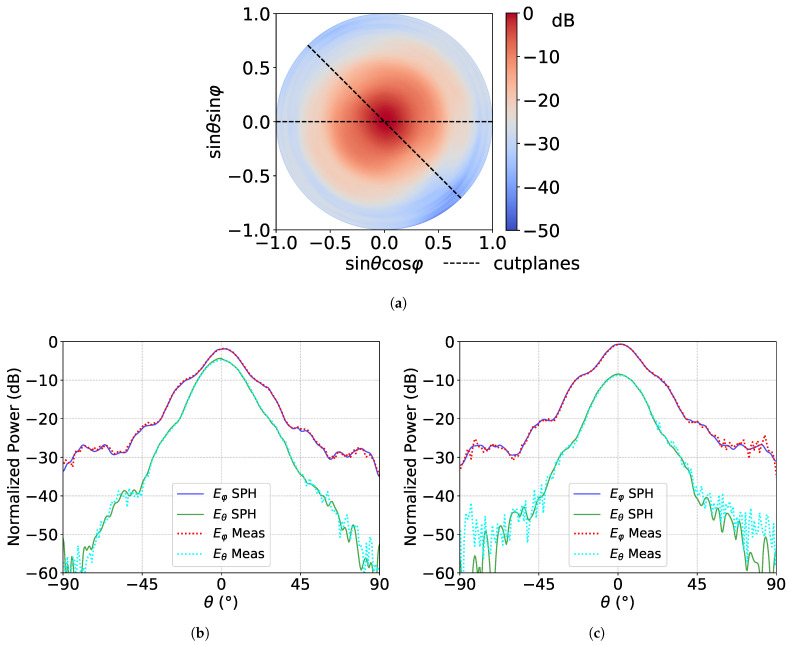
Far field radiation patterns of the circularly polarized horn antennas measured at 300 GHz. (**a**) 3D radiation pattern of the total field and (**b**,**c**) radiated fields in the cutting planes ϕ=0° and −45°, respectively, for both θ and ϕ components of the field, measured (Meas), and extracted by the spherical harmonic expansion (SPH).

Figure 5 displays the far field radiation patterns. There is an excellent agreement between the 2D patterns of reference and the field extracted from the 3D measurement that validates both the fast 3D pattern measurement approach and the phase measurement. Let us stress that this test case is a challenging one, since it combines a moderate directivity and metallic parts, leading to diffraction effects at low angles. Therefore, ripples in the low sidelobe levels (below −40 dB) and grazing angles, are inevitable for direct measurement, and they are partially filtered thanks to spherical harmonic expansion.

**Figure 6:**
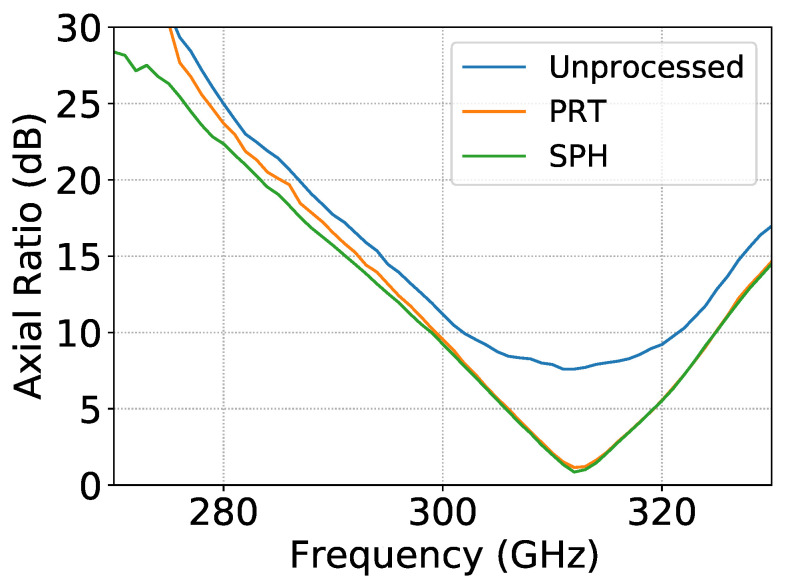
Axial ratio of the circularly polarized horn as a function of the frequency. The axial ratio is extracted from the raw measurement data (unprocessed), measured according to the polarization rotating technique (PRT) and deduced from the spherical harmonic expansion (SPH).

Moreover, the axial ratio of the antenna is characterized as a function of the frequency, see Figure 6. The computation of this metric requires the use of both magnitude and phase of the field and is therefore a valuable metric to assess the measurement of the phase. The axial ratio is measured according to two independent approaches: the polarization rotating technique and the one that is extracted from the spherical harmonic expansion. The excellent agreement demonstrates that the phase is accurately measured. The discrepancies between these axial ratio and the one that is derived from the raw (unprocessed) data clearly show the success of the error compensation procedures.

### 3.3. Multibeam Pillbox Antenna at 270 GHz

**Figure 7:**
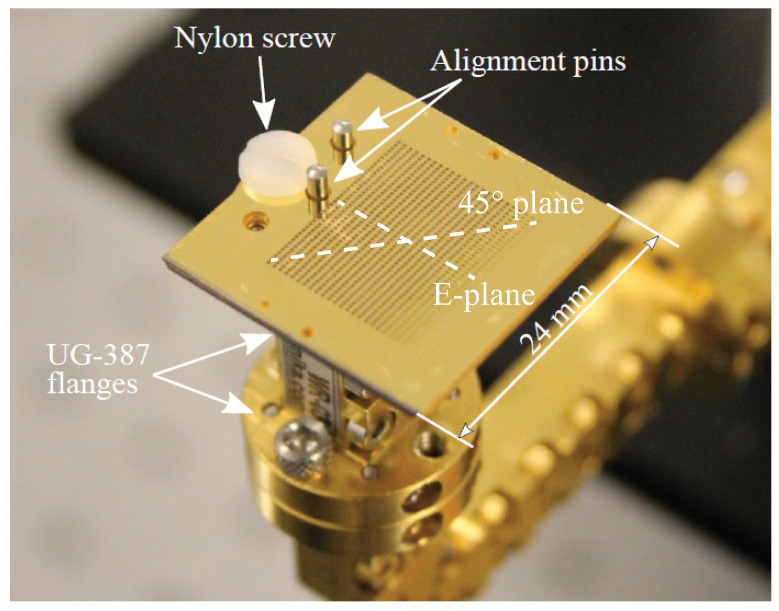
Picture of the pillbox antenna designed by KTH and IETR [28]. The operation bandwidth spans from 220 to 300 GHz.

The AUT is a pillbox that has been designed by KTH and IETR [28] to work between 220 and 330 GHz. The antenna, as shown in Figure 7, has been manufactured by KTH and measured at IETR. Similarly to a leaky wave, the AUT has a frequency beam scanning behavior, which requires 3D measurements for a proper characterization. More details about this antenna are provided in [28].

**Figure 8:**
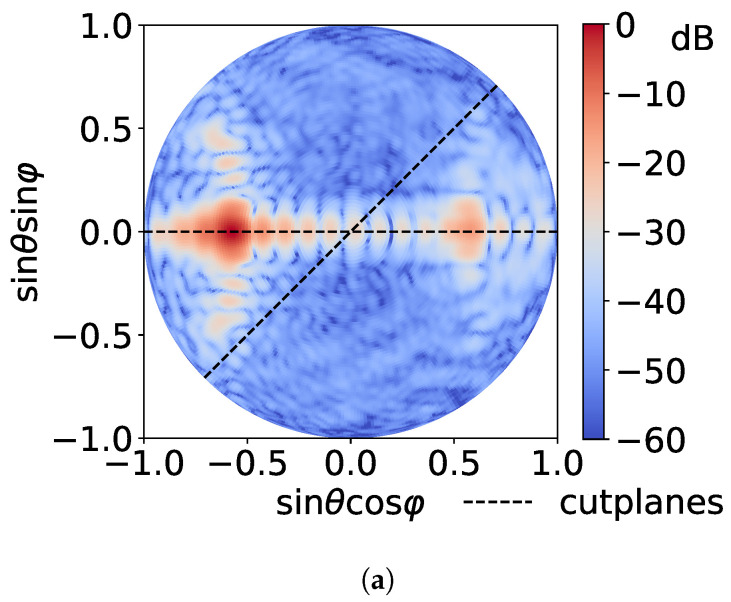

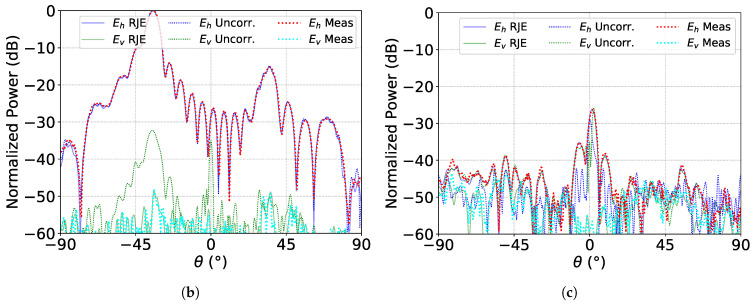
Far field radiation patterns of the pillbox antenna designed by KTH and IETR [28] measured at 270 GHz. (**a**) 3D radiation pattern of the total field and (**b**,**c**) radiated fields in the cutting planes ϕ=0° and ϕ=45°, respectively, for both horizontal and vertical components of the field. The measured field of reference (Meas) is plotted with the one extracted from the spherical harmonic expansion with and without RJE correction (RJE and Uncorr, respectively).

Figure 8 displays the far field patterns of the AUT. A tracking is performed to measure the radiated field over the reference cutting planes according to the horizontal and vertical components. The 3D field is first measured according to both θ and ϕ components, and then transformed into vertical and horizontal components following Ludwig 3 formalism. Cutting planes show the significant impact of the RJE correction, leading to an excellent agreement between the measured field and the one reconstructed via spherical harmonic expansion after RJE compensation, even at low magnitude levels. It means that not only the amplitude, but also the phase of the far field is properly measured in the sub-millimeter frequency range.

## 4. Conclusions

Procedures to estimate and compensate measurement errors that arise from the submillimeter wave range have been proposed and successfully applied to characterize antenna far field patterns at terahertz. At these frequencies, small mechanical and/or electrical errors, such as the ones inevitably brought by rotary joints and cables, are sufficient enough to significantly corrupt the measurement of the phase and, therefore, degrade the characterization of antenna 3D patterns.

The 3D far field patterns of two radiating structures have been measured in the submillimeter wave range. The agreement between the 3D and 2D patterns of reference as well the discrepancies between raw and compensated data experimentally demonstrate the validity of the proposed error mitigation approaches. The proposed procedures are applicable to both direct far field measurement facilities and CATRs.

## Data Availability

Not applicable.
